# Abstracts from German Journals

**Published:** 1873-05

**Authors:** Ch. Rauschenberg

**Affiliations:** Atlanta, Ga.


### ABSTRACTS FROM GERMAN JOURNALS.

BY CH. RAUSCHENBERG, M.D., ATLANTA, GA.

### *ON THE CATARRH OF THE GENITAL ORGANS OF THE FEMALE—(FLUOR ALBUS)*

[CONTINUED FROM MARCH NUMBER.]

While the catarrhal affections of the womb, which we have been discussing in the preceding pages, interfere seriously with the general health, destroy the faculty of conception, and occasionally lead to carcinoma, those of the vagina, to which we now direct our attention, may exist for years without producing nervous derangements or deeper destructive alterations; and only where the discharge connected with them is very copious, they interfere with the general health by this continued drain of material from the system.

The primary, uncomplicated vaginal catarrh is very rare ; the secondary one, combined with a primary uterine, and particularly cervical catarrh, is a very common disease, either as a consequence of the continuation of the catarrhal irritation from the mucous membrane of the womb to that of the vagina, or of the irritation of the latter by the morbid secretions of the womb, or both, or as a part of a general catarrhal affection of the genital track.

Where the primary, uncomplicated vaginal catarrh exists, it is almost invariably the consequence of mechanical or chemical irritation in some form or another. Thus long retained and neglected pessaries, by mechanical friction, and by retention and stagnation of the secretions, are considered by our author very prominent instigators of that disease. Prolapsus of the vagina, frequent and excessive coition in public women, dust and cold air on the mucous membrane of the vagina, particularly in old females, where senile shrinking of the labia prevents perfect closure of the vagina, the irritating influences of decomposing urine in vesico-vaginal fistula, and above all the gonorrhœal infection, must be considered by the physician in every investigation of the origin of this disease.

Catarrhal inflammation of the introitus and vestibulum vaginæ are caused in the same manner, and in children often by ascarides finding their way into the vagina.

Dr. Hildebrandt, as an example of catarrh of the introitus vaginæ and vestibulum, gives the history of an excessively painful case of that kind, where a very copious acrid discharge, in spite of the most careful cleanliness, had caused extensive painful erosion of the labia majora and the upper part of the thighs, so that walking had become a torture. The subject was a fleshy lady, who had been married two years, but whose tense hymen on examination proved not ruptured, but expanded and elongated into a deep cul-de-sac reaching very nearly up to the cervix uteri. Cohabitation being impossible without the exercise of more or less force, and hence considerable tension of the parts to which the hymen is attached, consequently mechanical irritation produced a catarrh strictly confined to the introitus vaginæ and the hymen. Division of the same, and removal of the flaps, was followed by a speedy and perfect recovery.

In another case, a primary, profuse, purulent catarrh of the vestibulum was caused by the existence of numerous growths (molluscum simplex) of various sizes, and their friction against each other on the labia minora, the clitoris, etc. Removal of these growths, the consequences of too frequent and coarse coition, cured the leucorrhœal discharge.

Tumefactions and enlargements of the Bartholinian glands are a phenomena frequently accompanying catarrhal affections of the introitus vaginæ. These glands are situated immediately within the external lips, and send their short excretory ducts to the vagina, where they terminate immediately in front of the hymen. Obstruction of these excretory ducts by retained secretions, by shreds of epithelium, by dust, by swelling from mechanical irritation, are frequent complications of this form of vaginal catarrh. Careful catheterization of these ducts with fine probes at an early period will frequently drain off the stagnating secretion of the glands, remove the irritation, and prevent the inflammation and subsequent formation of abscesses within them and the surrounding tissue.

Another concomitant phenomenon of the catarrhs of the introitus, and sometimes a primary occurrence causing the catarrh, is a partial hypertrophy, principally of the papillæ of the mucous membrane of the urethra. In such cases we find the thick, swollen, bluish-red orifice of the urethra studded with several dark-red, easily bleeding, polypus-like tumors, seldom larger than a cherry, obstructing the passage of the urine to such an extent that generally a severe effort is required for its often very painful discharge. It causes much burning by moistening the sore surface of the vaginal entrance, and walking is only possible with the thighs kept apart; causing often, when forcibly attempted, inflammation, swelling and suppuration of the labia and clitoris, and always a copious, purulent, and often fœtid, secretion. Coitus is, even in mild cases, an impossibility on account of excessive pain and the diminished volume of the vagina by the swollen urethra. Forced coitus under such circumstances produces, generally, fainting of the female—vaginal hemorrhage often lasting for hours—and severe, painful inflammations, confining the suffering party to her bed for days. This class of cases might appear connected with gonorrhœal infection, but this is not often the case, as they occur more frequently in old decrepit females and in virgins after the menstrual period.

Lastly, the acute genital catarrh of females must receive a short notice. It occurs seldom primarily, but is mostly the consequence of an aggravation of the described chronic affections, caused by coarse external or internal insults. It occurs, however, occasionally as a primary disease. Severe and sudden exposures to cold during the menstrual period cause now and then extensive catarrh of the entire genital canal, with subsequent diffuse metritis and parametritis. It is sometimes one of the most annoying local phenomenon in typhus, where the profuse, thick, yellow, purulent discharge corrodes and irritates the labia, and gives rise, in the same way as in variola and scarlatina, to hard cicatrizations and narrowing of the vagina.

Milder forms of acute genital catarrhs, with but very little tumefaction and pain, but annoying sensations of heat and itching, are caused by leptothrix vulvæ and oidium albicans, the two forms of microscopic fungi sometimes developing themselves within the vagina. The most frequent form of acute catarrhal inflammation of the genital track is that caused by gonorrhœal infection—the acute gonorrhœa of females. Extensive swelling of the labia majora, redness, epithelial abrasions, œdema of the labia minora, the surface of the entire cavity of the vagina coated with a thin sanguino-purulent secretion, the mucous membrane œdematous, and, in consequence of epithelial losses, in many places of a bloody, streaky appearance, as if scratched, present themselves to the examining eye within the first three to four days of the affection. The introduction of the speculum is very painful, or impossible ; the urethra is frequently, but not always, at this period of the disease, considerably swollen, its orifice œdematous and covered with pus and blood. Burning pains in the abdomen, difficulty in walking, scalding and stinging sensations during micturition, are experienced from the commencement; and if additional causes of irritation, for instance dancing, the use of spirituous drinks, etc., are allowed to aggravate this state of things, we often see, as a consequence, a continuation of the inflammation to the mucous membrane of the uterus, and through that of the fallopian tubes to the ovaries; hence a more or less acute metritis and ovaritis, with pain in the corresponding hypogastric and lumbar region. The enlarged, heavily-movable and tender ovary begins to be perceptible, to the examining finger, within the vagina, and the patient is unable to stand erect or to move about; thus showing that neglected gonorrhœal inflammation of the female genitals can run a course analagous to the same disease in males, where, under the same noxious influences, the prostate gland, the canaliculi seminales, the epididymis and the testicle can become involved.

The acute gonorrhœal catarrh of females has still a greater inclination to become chronic, and to occupy a duration of years and months, than that of males, evidently on account of the greater extent and larger folds of the mucous membrane. Its diagnostic distinction from common catarrh in this protracted state becomes an impossibility, unless pointed condylomata and catarrhal affections of the urethra furnish a governing hint.

Prof. Hildebrandt at this occasion desires to call the attention of the profession to the fact that he has, in his extensive experience, met four times with a concurrence of circumstances which seemed to establish the fact that the papillary ulcerations of the external os of the uterus and of the cervix, which lead by proliferation of the papillæ so often to carcinoma, and which have been spoken of above, are most frequently occasioned by gonorrhœal infection, and follow that infection comparatively quick.

If future observations should determine that his observations have not been the result of accidental occurrences, and that there should exist a casual connection between gonorrhœa and papillary ulceration in an analagous manner as between gonorrhœa and the formation of condylomata on the external genitals, the old and almost forgotten ætiology of cancer of the womb, as a consequence of gonorrhœal infection, would be reëstablished.

While the affection which we have been discussing as fluor albus, or as catarrh of the female genitals, presents a variety of forms, its treatment is simple, and consists generally in a proper use of caustics and astringents, varying only in relation to the manner and strength of application; and while this is correct, generally speaking, many cases occur in which quite a complicated course of treatment becomes necessary, and the old habit of physicians to administer astringent vaginal injections in every leucorrhœal discharge of argentum nitricum or cuprum sulphur or tannin, etc., without previous careful examination of the genitals, and hence, without any conception of the indications to be fulfilied, deserves certainly no imitation.

Here Prof. Hildebrandt refers to the case of a delicate blonde, then an inmate of his hospital, who, although very young, is the mother of five children, has aborted three times, and has been suffering seven months—ever since the last abortion—with profuse fluor albus, without pain, but with general weakness and emaciation as consequences. She has taken preparations of iron, and used alum injections, at the advice of her former attendants, but the iron caused no improvement of nutrition and strength, and only increased menstruation; the injections caused finally only increased irritation of the uterus and bladder, without checking the morbid discharge. A close inspection of the uterus revealed it enlarged and loosened in its texture. The probe slipped into a wide and flabbid cavity, causing pain and hemorrhage as soon as the blunt end of it came in contact with uneven but soft and compressible spots on the fundus. The cavity was dilated with laminaria tents, and the examining finger discovered at the fundus uteri an uneven, somewhat lobular mass, of the size of a bean. This being smoothly removed with the finger-nail from the surface of the membrane, and a small quantity of diluted muriated tincture of iron injected, the secretion ceased almost entirely within two weeks, and the general health of the patient improved perceptibly.

This case illustrates the folly of astringent injections without regard to a closer consideration of the origin and cause of these cases, and demonstrates the leading features of the treatment of the catarrhs of the corpus uteri, which we will first discuss, to wit: *direct local treatment of the diseased mucous membrane and removal of any foreign bodies that may exist.* The foreign bodies occurring in that locality are initial sprouts of fibrinous polypi, as in the above described case, or only polypus-like excrescences following degeneration of the glands, or simple hypertrophies of the mucous membrane. The removal of all these commonly not very extensive exuberances can mostly be accomplished with the finger-nail. Instruments, like Recamier's curette, are never used by Prof. Hildebrandt. He holds that serious defects may be caused by their sharp edges in the healthy portions of the womb, while no certainty exists that the morbid portions are removed completely at the same time. If, after the removal of the morbid growths the catarrh does not cease, the proper local treatment of the mucous membrane must be instituted.

Our author does not enter into a detailed critical analysis of the different methods of local application of remedies to the mucous membrane of the cavity of the womb, but states pointedly and short, *that he can not advocate their introduction into that cavity in a solid state ;* that he considers the introduction of solid nitrate of silver, even in very small portions, as unreliable and dangerous as it is painful ; that the application of bacculi of tannin or alum and gum arabic is not less injurious ; and the introduction of pledgets of lint, saturated with astringent solutions, a senseless proceeding. Whether the cauterizations of the cavity of the womb by the porcelain burner of the galvano-caustic apparatus will prove as safe as Prof. Spiegelberg, whom he esteems a very reliable gynæcologist, has found it, the future must yet teach ; hence Prof. Hildebrandt considers *no method of local application of remedies to the cavity of the womb safe, but the injection of proper fluids under proper precautions and with suitable instruments.*

The usefulness and safety of his method of making intra-uterine injections he claims to be a fact well known to every one of the students from his obstetrical clinic, where he uses them in profuse post-partum hemorrhage in weakening catarrhal discharges, in diptheritis, in putrid decomposition of retained placental portions or blood-coagula, etc. The first efforts to treat the non-puerperal diseased womb by injections were often followed by the most fatal results—as metritis, peritonitis and death—and only the experience of the last ten years has revealed the proper precautions by the observation of which uterine injections become as safe as they are curative and salutary. Before stating these precautions, and his method of making intra-uterine injections, Prof. Hildebrandt mentions that he speaks only of catarrh proper, or of that morbid condition of the mucous membrane following in a flabby uterus the puerperal state and abortions, etc., but not of those secondary catarrhal affections which accompany the hypertrophic uterus, indurated, or, still worse, surrounded by extensive exudations. These conditions do not bear the use of injections.

Only warm fluid, from ten to fifteen drops, and only weak solutions, until the irritability of the uterus has been tested and the necessity for stronger remedies become plainly established, must be used to avoid painful uterine colic and other still more distressing consequences. Solution of sesqui-chlorate of iron, tincture of iodine and glycerine, the two former in different dilutions, but sometimes entirely undiluted, are the only remedies he recommends for these injections. All other caustics are either not sufficiently tested or abandoned as injurious. The glycerine is the most useful in cases of flabby chronic swelling of the mucous membrane prominently connected with the profuse catarrhal discharges of old women and chlorotic girls. Its usefulness consists in its dessicating effect. The most strenuous efforts of the advocates of uterine injections of late, however, have *principally and justly been directed to the discovery of the most suitable method to insure speedy and complete discharge of the injected fluid from the cavity of the womb.*

English physicians have, for this purpose, invariably dilated the cervix by laminaria or sponge tents, in order to bring on the same favorable condition which exists in the womb during the puerperal state ; and German physicians, with very few exceptions, have followed their example. One portion of those who do not dilate but inject at once, use only three, five or eight drops, which they allow to remain within the cavity ; another portion of them inject large quantities, and endeavor to accomplish the discharge of the injected fluid by the use of a double tubed catheter. Both methods are not sufficient to avoid all the dangers connected with intra-uterine injections ; the much praised method of previous dilatation can not be recommended, as it is not always safe, but always much more tedious and painful than Prof. Hildebrandt's method. He injects, according to the capacity of the cavity, a quantity of from ten to twenty drops, with Braun's uterine syringe ; allows it to remain in one or more seconds, until he believes it has exercised its influence on the diseased membrane, and then withdraws it again into the syringe by the retrograde movement of the piston. In order to take up all the injected fluid, he gradually draws the canule of the syringe from the internal towards the external os of the cervix, in correspondence with the retrograde movement of the piston. This method has been used by him for a number of years, and he recommends it as perfectly safe, and much more appropriate and effective in many cases than the dilatation by tents. After one of his injections, thus executed, the patient need not remain in the recumbent posture longer than one-half an hour; after it she can move about freely, and in two or three days the injection can be repeated. The preparation of the uterus by the tents for the purpose of making uterine injections requires hours, sometimes days, and sometimes—as considerable dilatation is essential in order to accomplish the opening of the internal os for a sufficient length of time to insure efflux of the injected fluid—a single dilatation is not sufficient, but a second and third repetition becomes necessary. Thus, for the accomplishment of one intra-uterine injection twenty-four to thirty-six hours confinement to bed are unavoidable, and this process is repeated for several weeks every fourth, fifth or seventh day—certainly a procedure connected with much torture and exertion on the part of the poor patient. In addition to this, the repeated introduction and removal of the sponge tents, independent of the pain which it causes, affects the womb unfavorably. The mucous membrane of the cervical canal is not only more or less injured by pressure, but it is partly destroyed, and the internal oriffce becomes, sooner or later, more or less irritable; and to this irritated orga , partly deprived of its mucous membrane, the injection is administered. It is obvious that under such circumstances even weak solutions may act too severely, and, as injections in order to cure the catarrh must be repeated, they may under these circumstances produce metritis. Finally, there exists no absolute certainty that this method really answers the purpose for which it is instituted, to wit, to secure speedy efflux of the injected fluid. It is well known how rapid the dilated internal orifice contracts in an irritable womb; and this will certainly take place so much quicker when the injected fluid exercises an abnormal degree of irritation in the region of the internal orifice.

None of these objections can be urged against the above described method of Prof. Hildebrandt.

It must be well remembered, however, that local treatment does only answer the purposo in those catarrhs of the uterine cavity which have been produced by purely local causes. Where constitutional causes have occasioned the disease, these, as well as some other concomitant phenomena—for instance disturbance of digestion, of sleep, of the cutaneous functions, etc.—must receive special consideration and treatment, according to the accepted therapeutical principles of internal medicine.

This same rule applies with equal correctness to the catarrhs of the cervical canal. A number of them require only a very careful general treatment, and very energetic local interference may prove absolutely injurious. This is particularly true of the cervical catarrh of females affected with phthisis pulmonalis. Caustic and astringent applications almost always cause an increase of the local trouble, the cervix continues to swell, the cauterizations become more and more painful, the quantity of the morbid secretions continues the same or increases. The fact that under such circumstances the local treatment does more harm than good can not be denied, because it weakens the patient physically, and by its ineffectiveness still lessens the already diminished confidence of the patient in her recovery. Every patient with cervical catarrh, who does not present the image of fair general health, ought to be carefully examined in relation to the condition of her lungs, and even a strong suspicion of tubercular affection should restrict the local treatment of the womb to mild, demulcent injections, such as tepid decoction of linseed infusion of German chamomile, etc.

The same is true of scrofulous subjects, and a long continued general treatment, consisting in cool bathing, the internal use of mineral waters containing iodine and iron, with mere local cleanliness of the genitals, should precede all energetic local measures.

The local treatment of the cervical catarrh of females consists, prominently again, in the use of astringents and caustics, which are applied either to the ulcerating or otherwise degenerating lips of the cervix or to the mucous membrane of the cervical canal, in a more or less concentrated form. Their method of application is materially simpler than it is when applied to the uterine cavity, and the differences between virgins and females who have had children establish the principal differences of treatment.

In virgins, as has been stated above, the cervix is generally found pointed, the orifice narrowed by the formation of cicatrices, the canal of the cervix obstructed by swelling of the folds of the mucous membrane, hypertrophy of the glands and inspissation of the secretion. The first indication in these cases is *ample dilatation of the cervical canal to allow the secretion to flow off freely, and to admit the direct application of caustics to the diseased mucous membrane.*

The application of dilating tents does not fulfill this indication, because within a few days after their removal the cervical canal contracts again to its original size, and in spite of every new dilatation the disease returns again. Only an ample division of the walls of the cervix, on both sides, with the knife can accomplish a lasting dilatation in these cases. A complete cicatrization of the edges of the womb must have taken place, and that is generally the case within two to three weeks before any cauterization of the mucous membrane can be instituted.

The cervix thus transversely divided by the knife, or naturally of that shape in those females who have had children, bears severe cauterization, provided it is not extended higher up than below the internal os. Cauterization of that portion is not advisable, as it reacts intensely even against moderate irritation, so that a violation of this precaution may easily be followed by diffuse metritis and parametritis. The selection of the caustics to be used depends much upon the duration of the disease and the depth of the degeneration of the mucous membrane. In general those caustic remedies whose effect as to depth can not be well calculated are avoided—to wit, the alkaline caustics. In mild cases Dr. Hildebrandt recommends the use of tannin bacculi, made of tannic acid, and as little gum arabic and glycerine as possible to shape it into small staffs for introduction into the cervical canal. A piece the length of one centimeter is introduced with the index and middle finger of the right hand, or with a pair of curved polypus forceps through the speculum, and remains there to be dissolved. If they do not prove sufficient to change the morbid condition of the mucous membrane the solid stick of nitrate of silver is resorted to.

It is introduced along the cervical canal, within the neighborhood of the internal orifice, allowed to remain a few seconds, and then withdrawn. The tannin bacculi, as well as the nitrate of silver, are repeated at intervals of seven days. In cases where the papillæ have already commenced to exuberate, where hypertrophy of the glands and of the mucous membrane in toto exists, even the nitrate of silver does not suffice, and then an early application of the actual cautery constitutes the main reliance of Prof. Hildebrandt. A long and ample experience has forced him to the conviction that the immediate and early use of this measure, in cases of that character, avoids the unnecessary delay of a cure always connected with the use of less energetic means of cauterization. Where the objections of the patient prevent the use of the actual cautery, he substitutes the chloro-acetic. It acts approachingly as powerful as the other; causes the formation of a firm scurf without penetrating to an incalculable depth into the tissues, as the chromic acid so highly recommended by Sims is inclined to do. Its application is performed with the aid of a glass staff, but its effect is not so even and uniform as that of a conical or olive-shaped cautery.

Whenever the cervical canal returns to the healthy condition —which the decrease of its dimensions, the width of its cavity, and its glassy, purulent secretion indicates—the ulcerations of its lips, which are unavoidably cauterized, with the cavity cicatrize speedily at the same time. If ulcerations from glandular hypertrophy, or of a purely catarrhal character, remain on the lips, which, when cleanliness and rest are neglected, is sometimes the case, slight touches with nitrate of silver must be made in intervals of from five to seven days. Papillary ulcerations, which frequently bleed under the use of lunar caustic, yield much better to applications of chromic acid, tincture of iodine, muriated tincture of iron, or pyroligneous acid. It is important, however, to cease with these severe cauterizations for a longer period as soon as the edges of the ulcerations become smooth and show a disposition to heal. Mere ablutions of the diseased spots with very mild solutions of lunar caustic, sulphate of copper, creosote water, solution of alum, etc., are then generaliy sufficient to complete a cure.

The same remedies which are used in the cervical catarrh are generally sufficient for the treatment of the catarrhs of the vagina and the vestibulum.

In the acute gonorrhœa of females, after the inflammatory stage—under the use of absolute rest in the recumbent position, warm diluent injections, and, perhaps, warm hip baths—has run its course, the application of first very energetic and afterwards milder and milder cauterizations constitutes the main treatment.

An acute purulent catarrh can be arrested most effectually and speedily by careful cauterization of the entire mucous membrane of the vaginal cavity, from the cervix uteri down to the introitus vaginæ, with the solid nitrate of silver by the aid of the speculum. The same method is used by Prof. Hildebrandt in the numerous and obstinate vaginal catarrhs of old women, in the vaginitis adhesiva and the profuse catarrhs of the vestibulum. After the first copious discharge has been arrested in this manner, and a thin, less purulent secretion continues to flow from a still loose mucous membrane, free ablutions of the vagina with the above mentioned fluids, by the aid of the speculum, are continued until the cure is complete.

If the catarrh becomes chronic, but remains otherwise uncomplicated, the application of a tampon of the size of a hen-egg, covered with an ointment consisting of five parts of alum and thirty of adeps, is a most effectual remedy. Females learn very soon to introduce these tampons themselves. They should be retained without the aid of Simpson's Tampon Supporter, as that instrument by mechanical irritation frequently occasions sexual excitement, and is therefore condemned by Prof. Hildebrandt. Equally effectual is the glycerince tampon, which Sims has brought into extensive use. Its usefulness becomes principally apparent in simple vaginal and cervical catarrhs without ulceration, such as occur after child-bed from deficient involution of the genitals, and the beneficial results obtained by their use depend on the dessicating effect of the glycerine upon the tissues. They are only worn in the night, as the copious discharge going on while they are in contact with the parts would keep the underclothing of the wearer unpleasantly moist, and expose her to the risk of taking cold in day time.

Besides these remedies and modes of treatment, there are many others used by other gynæcologists, but those discussed in this article are such as have been amply tested by our experienced author, and have been found by him reliable and safe.

A very accurate general examination, a minute consideration of the constitutional peculiarities and of the condition of the entire genital canal and its surroundings, in every case, constitute an indispensible requirement for a successful treatment of this disease.—*Volkman's Sammlung Klinischer Vortrage.*

